# Bioreactor Operating Strategies for Improved Polyhydroxyalkanoate (PHA) Productivity

**DOI:** 10.3390/polym10111197

**Published:** 2018-10-26

**Authors:** Warren Blunt, David B. Levin, Nazim Cicek

**Affiliations:** Department of Biosystems Engineering, University of Manitoba, Winnipeg, MB R3T 5V6, Canada; umbluntw@myumanitoba.ca (W.B.); nazim.cicek@umanitoba.ca (N.C.)

**Keywords:** bioreactor, continuous fermentation, fed-batch fermentation, PHAs, polyhydroxyalkanoates, productivity, scale-up

## Abstract

Microbial polyhydroxyalkanoates (PHAs) are promising biodegradable polymers that may alleviate some of the environmental burden of petroleum-derived polymers. The requirements for carbon substrates and energy for bioreactor operations are major factors contributing to the high production costs and environmental impact of PHAs. Improving the process productivity is an important aspect of cost reduction, which has been attempted using a variety of fed-batch, continuous, and semi-continuous bioreactor systems, with variable results. The purpose of this review is to summarize the bioreactor operations targeting high PHA productivity using pure cultures. The highest volumetric PHA productivity was reported more than 20 years ago for poly(3-hydroxybutryate) (PHB) production from sucrose (5.1 g L^−1^ h^−1^). In the time since, similar results have not been achieved on a scale of more than 100 L. More recently, a number fed-batch and semi-continuous (cyclic) bioreactor operation strategies have reported reasonably high productivities (1 g L^−1^ h^−1^ to 2 g L^−1^ h^−1^) under more realistic conditions for pilot or industrial-scale production, including the utilization of lower-cost waste carbon substrates and atmospheric air as the aeration medium, as well as cultivation under non-sterile conditions. Little development has occurred in the area of fully continuously fed bioreactor systems over the last eight years.

## 1. Introduction

The production of completely biodegradable materials from renewable resources is an urgent challenge for the mitigation of the cradle-to-grave environmental impacts associated with petroleum-based plastics. In 2015, the worldwide production of synthetic plastic materials exceeded 320 million tonnes [1], and at current consumption rates, it is estimated to reach 33 billion tonnes by 2050 [2]. Of the amount currently produced, less than half is recycled or landfilled [2]. An astonishing amount of these materials are unaccounted for, and persist in natural ecosystems due to their high molecular weight and recalcitrance to biodegradation [3,4,5], which can have multiple detrimental impacts on both terrestrial and aquatic ecosystems. It is estimated that 10 to 20 million tonnes of plastic material is discarded into oceans alone [6]. Once in the natural environment, plastics can absorb and concentrate persistent organic pollutants [7], and release toxic additives that are not chemically bound in the polymer [8,9]. Through mechanical abrasion, plastics are broken down into microsized to nanosized particles that bioaccumulate in filter-feeding animals, posing a significant threat to marine ecosystems [6]. Recent evidence suggests that particles <10 μm may pose significant risks to human health through inhalation and the penetration of pulmonary surfaces [10,11].

Microbial polyhydroxyalkanoates (PHAs) are a class of bio-based and biodegradable polymers that may help displace certain applications for traditional plastic materials [12,13,14]. PHAs are intracellular carbon and energy storage polymers that are synthesized (generally under growth-limiting conditions) by more than 300 species of Gram-positive and Gram-negative bacteria [13,15,16], as well as several species of archaea [17]. These R-configuration hydroxyalkanoic acids have properties that are often compared to polyethylene or polypropylene [18,19,20], but can be completely broken down to water, biomass, and CO_2_ through the enzymatic activity of microorganisms [21]. On the basis of the length of the monomer subunits, PHAs are generally classified as either short-chain length (scl), or medium-chain length (mcl). The scl-PHAs have monomer subunits with carbon chain lengths of three to five carbon atoms. To date, the most common and best characterized PHAs are poly(3-hydroxybutryate) (PHB) and copolymers of PHB with poly(3-hydroxyvalerate) (PHBV) [19,22]. In contrast, mcl-PHAs have carbon chain lengths of six to 14 carbon atoms, and account for 95% of the 150 identified PHA monomers, implying the potential for many polymers with different properties and a diversity of applications [23,24]. For the remainder of this document, all of the abbreviations referring to the PHA composition will imply the 3-hydroxy form, whereas the 4-hydroxy form will be specified explicitly (i.e., P(4-HB)). 

Life cycle analyses have shown that PHAs, depending how they are produced, can outperform polyethylene or polypropylene in impact categories that include CO_2_ production, ecological footprint, and environmental toxicity [25,26,27], as well as global warming potential and non-renewable energy consumption [28,29,30]. However, several reports have indicated that the production of PHA requires more fossil fuels than petrochemical plastics [31,32]. The impact appears to be largely dependent on the available carbon feedstock as well as the nature of the electricity source that is used in the production process. Despite mixed reports on life cycle analysis, there is still much optimism around PHAs, and it is anticipated that as production technology evolves, life cycle analyses will increasingly favor PHAs [33,34]. 

Despite this promise, PHAs represent only a very small fraction of global biopolymer production, which in turn represents at most 5% of the current global plastic market [27,35]. Only a few companies currently produce PHAs at pilot-scale or industrial-scale levels [25,36], and their application is limited to a few niche markets. A major factor for this is the high production cost of PHAs, which is estimated to be anywhere from three to 15-fold higher than polyethylene or polypropylene [36,37]. The cost of the carbon source accounts for a large portion (most estimates are 40–50%) of PHA production costs [38,39,40]. Much recent focus has been given to identifying inexpensive and readily available carbon substrates for PHA synthesis [38,39,41,42,43,44].

In addition to using low-cost carbon feedstock, efficient cultivation techniques aimed at obtaining high rates, yields, and product titers are crucial to the sustainability of PHAs [38,45]. The objective of this review is to summarize past studies regarding bioreactor operation strategies for high PHA productivity. These include a variety of fed-batch, cyclic batch, or cyclic fed-batch processes (semi-continuous), and fully continuous bioreactor systems. In addition, where possible, we also examine the degree to which these technologies have been scaled-up to pilot or industrial levels. Although the primary focus of this article is given to mcl-PHAs, the literature on scl-PHAs is often referred to as a useful point of reference. 

The terms that are used throughout this paper are defined here, including: total biomass, [*X_t_*] (g L^−1^); PHA biomass, [*X_PHA_*] (g L^−1^); non-PHA residual biomass, [*X_r_*] (g L^−1^); intracellular PHA content, *%_PHA_* (expressed as a percent of cell dry mass, CDM*)*; volumetric productivity, *Q_v_* (g L^−1^ h^−1^); and yield of PHA from the carbon substrate *Y_PHA/S_* (g g^−1^ or mol mol^−1^). Where appropriate, PHB may be used interchangeably with PHA in these terms for improved clarity. Square brackets are used to indicate concentration.

## 2. Production of PHAs in Fed-Batch Bioreactor Systems

While batch cultivations are useful in certain bioprocessing applications or to study physiology, they are not ideal for high-productivity PHA cultivation processes. In batch cultures, the reactants (i.e., carbon, NH_4_, PO_4_^3−^, Mg^2+^) are added initially without any further addition during the cultivation process, so the initial concentration is restricted to sub-inhibitory levels. Due to this, the cell density and productivity is limited, and batch cultures are therefore not discussed in this review, but have been reviewed recently elsewhere [46]. The objective of applying fed-batch cultivation to PHA production is to combine high cell density (HCD) cultures with high *%_PHA_* in order to maximize *Q_v_*. Fed-batch cultures are usually started in batch mode, and the culture is fed with nutritional components for growth and/or PHA synthesis in order to prolong the exponential-phase growth at a high rate, thereby maximizing cell density as quickly as possible [47]. To date, HCD fed-batch cultures have demonstrated the highest *Q_v_* in both scl-PHA and mcl-PHA production [48,49], and are also used in most industrial production settings [50].

Obtaining high *%_PHA_* is also a very important factor for high *Q_v_*, improving the ease of downstream processing, and overall process economics [51,52]. For most of the production strains in which PHA synthesis is not growth-associated, the growth phase is followed by continued carbon feeding in the absence of another nutrient, which is often NH_4_ or PO_4_^3−^, to promote high *%_PHA_* at the time of harvest. Thus, the cultivation process is usually divided into a growth phase and a PHA accumulation phase, with both occurring in the same vessel, but separated temporally, although this is not always the case. For example, some organisms such as *Azohydromonas lata* and recombinant strains of *Escherichia coli* (recombinant implied hereafter with respect to *E. coli*) are known to accumulate PHA concurrently to growth, although in *A. lata*, the application of N-limitation reportedly improved PHA synthesis [53,54]. 

Other studies have shown that mcl-PHA production occurs concurrently to growth when the carbon uptake rate is limited by the feeding rate, such that nutrient limitation is unnecessary [55,56]. However, generally, the application of nutrient limitation is known to be beneficial to PHA synthesis, and the timing has been shown to be important [57,58] for the general case where growth and PHA synthesis occur in separate phases. Fed-batch cultivations can also be used to improve the *Y_PHA/S_* for a given substrate. This is usually done by supplying a lower-cost carbon source (often glucose) for cell production during the growth phase, with subsequent feeding using substrates that typically exhibit high *Y_PHA/S_*, but are often more expensive. For example, Sun et al. obtained a *Q_v_* of 1.44 g L^−1^ h^−1^ using *P. putida* KT2440 when feeding nonanoic acid at an exponential growth rate that was designed to maintain a specific growth rate (μ) of 0.15 h^−1^ [55]. When nonanoic acid was co-fed with glucose (1:1 mass basis) at an exponential growth rate (μ = 0.25 h^−1^) followed by linear feeding, a very similar *Q_v_* was obtained, but the yield of PHA from nonanoic acid improved from 0.60 g g^−1^ to as much as 0.69 g g^−1^ [56]. Similar strategies have been applied by others [59,60,61,62,63]. 

Several high-productivity fed-batch cultivations using pure cultures are shown in Table 1 for mcl-PHA and Table 2 for scl-PHA studies. Most of the mcl-PHA studies have used relatively expensive mcl-fatty acids to obtain *%_PHA_* as high as 76% CDM [59], *Q_v_* as high as 2.13 g L^−1^ h^−1^ [48], and *Y_PHA/S_* as high as 0.86 g g^−1^ [60]. Although glycerol and carbohydrate-based substrates are not generally associated with high *%_PHA_* [42,64,65,66], a few studies have obtained *%_PHA_* as high as 67% CDM from glucose and 47% CDM from glycerol [67,68] using a recombinant strain of *P. putida* KT2440. Another study obtained 63% CDM as mcl-PHA from an equimolar mixture of glucose and fructose using *P. putida* IPT046 [69].

Despite some promising results for fed-batch mcl-PHA cultivations, as shown in Table 1, a comparison with Table 2 shows that, in general, the *Q_v_* of mcl-PHA cultivations is low when compared with scl-PHA production processes. Ryu et al. [84] reported achieving [*X_t_*] of more than 280 g L^−1^ CDM with [*X_PHB_*] of more than 230 g L^−1^ (*%_PHB_* = 83% CDM) using *C. necator* grown on glucose. While these are the highest known experimental values for [*X_t_*] and [*X_PHA_*], Wang and Lee [49] reported the highest *Q_v_* (5.13 g L^−1^ h^−1^) using *A. lata* (formerly *Alcaligenes latus*) cultured on sucrose. By comparison, MacLean et al. [48] have reported the highest mcl-PHA *Q_v_* from inoculation to harvest at 2.13 g L^−1^ h^−1^. As shown in Table 1, the [*X_t_*] seldom exceeded 100 g L^−1^ in mcl-PHA cultivations, and only one study has reported [*X_PHA_*] of more than 70 g L^−1^ [75]. Generally, mcl-PHA cultivations appear to be characterized by lower [*X_t_*] and lower *%_PHA_*. The reasons for the productivity discrepancy between scl-PHA and mcl-PHA production remains unclear, but the *%_PHA_* might be explained by differences in granule morphology [21,72].

Interestingly, the number of studies targeting cultivation strategies for achieving the highest possible *Q_v_* appeared to peak in the mid-1990s for scl-PHAs, and perhaps 10–15 years later for mcl-PHAs. While these studies are valuable tools for understanding process engineering design and limitations, they are hindered by a few key impracticalities. These include the use of high-purity carbon substrates as well as using O_2_-enriched air for aeration. More recently, there appears to be somewhat of a shift toward PHA production from waste substrates such as crude glycerol [90,99], plant-based oils [77,79,94,95,102], and lignocellulose-based hydrolysates [98], and devising strategies to achieve HCD cultures without use of purified O_2_ streams [61,62,103].

### 2.1. Control of Fed-Batch Cultivations

A significant challenge in fed-batch cultivation is predicting the nutritional requirements of the culture in real-time so that an appropriate feeding strategy can be implemented. Direct and rapid measurement of the parameters of interest (i.e., [*X_t_*], *%_PHA_*, residual concentrations of carbon, NH_4_, PO_4_^3−^, Mg^2+^, etc.) is often not possible, or the existing methods require expensive analytical equipment with a considerable time delay [104,105,106]. Nonetheless, some studies have used online or rapid offline measurements of residual carbon levels using analytical techniques such as gas chromatography (GC) [73,82], high-performance liquid chromatography (HPLC) [89,91], and mass spectrometry (MS) [81]. Others have made use of online glucose analyzers [57,63,84,88,107].

Most often, feeding rates are predicted indirectly through using other parameters that can be rapidly and reliably measured using standard bioreactor equipment. Some of the more popular methods involve the use of off-gas CO_2_ measurements [79,80,107,108], dissolved oxygen (DO) measurements [49,67,77,109], pH measurements [53,54], optical density [58,75], and combinations thereof [58,75,101]. In general, with these approaches, carbon limitation results in reduced oxygen demand, which manifests itself as a large drop in CO_2_ production, reduced agitation rates, and also a rise in the DO signal. A rise in pH may also be detected with the exhaustion of the carbon source, assuming a negligible impact on pH from the production of acetate, amino acids, and the source of nitrogen [76]. With these methods, the carbon source is often fed pulse-wise upon detection of a limitation in order to bring the concentration back up to the desired level [49,53,58,86,94,95,98,102,110]. Interestingly, it appears that the chosen method of carbon addition can influence PHA production. For example, Możejko and Ciesielska [110] showed a significant increase in *%_PHA_* (from a maximum of 25% CDM to 44% CDM) in *Pseudomonas* sp. G101 when the carbon source (waste canola oil) was fed to the bioreactor by a pulse-feed strategy in comparison to a continuous drip-feed addition of an equivalent aliquot of waste canola oil. Although this type of feast–famine process is often applied to PHA-storing populations in mixed microbial cultures [40], these results may suggest that small-scale feast–famine is favorable for PHA storage in pure cultures. 

Alternatively, a more proactive strategy may be applied in which the culture is fed in order to maintain a predetermined (typically exponential) growth rate. This method has been quite successful in several studies, and has achieved [*X_t_*] nearing 100 g L^−1^ CDM for growth of *Pseudomonas* sp. on both fatty acid and carbohydrate-based carbon sources [48,61,62]. Typically, these models require the initial [*X_t_*], the change in μ over time, as well as establishing constant [59], linear [60,61], or decaying [48,72] feeding rates during the PHA accumulation phase (see Table 3). According to MacLean et al. [48], sudden decreases in feeding rates result in cell lysis and foaming, so it is important to make the transition between different stages of feeding smoothly. Several feeding strategies have been investigated, and are shown in Table 3.

Significant efforts have gone into developing various models that allow the culture to grow as quickly as possible at the start of the process, before curbing the feeding rate to match the transient carbon uptake rate as the culture transitions to PHA storage. Sun et al. [55] have shown that controlling the growth rate via the rate of carbon addition allows higher [*X_t_*] to be obtained before the process must be terminated due to inadequate oxygen mass transfer. Since lower growth rates are used, there is a trade-off in biomass productivity, but the constant carbon limitation is reported to cause simultaneous PHA accumulation and eliminates the need for the limitation of another nutrient to accumulate PHA. The onset of oxygen limitation inevitably limits *Q_v_* in HCD cultivations for PHA production and causes uncontrollable foaming and accumulation of the carbon source to potentially toxic levels (depending on the feeding strategy) [55,56,62,75,109,111].

### 2.2. Limitations of HCD Cultivations: The Oxygen Transfer Issue

Perhaps the biggest challenge with the control of HCD fed-batch cultures is maintaining an adequate supply of dissolved oxygen (DO). Gas-to-liquid mass transfer is driven by the concentration gradient between the liquid phase (*C_L_*) and the equilibrium concentration (C_L_*), the liquid film mass transfer coefficient (*k_L_*, i.e., distance time^−1^), and the interfacial area between the gas and the liquid (*a*), as described in Equation (1) [113]:

(1) $$\frac{dDO}{dt}=k_{L}a\left(C_{L}^{*}-C_{L}\right)$$

As shown in Equation (2), if *k_L_a* is known, the oxygen uptake rate (OUR) of the culture can be estimated from the rate of change of DO with respect to time. Assuming no buildup of O_2_ in the liquid phase (i.e., steady-state conditions) the OUR can be assumed to be equal to the oxygen transfer rate (OTR), which can be written as shown in Equation (2) [114]:

(2) $$\mathrm{OUR}\approx \mathrm{OTR}=k_{L}a\left(C_{L}^{*}-C_{L}\right)$$

With the onset of O_2_ limitation being problematic in strictly aerobic bioprocesses such as PHA production, many studies have focused on enhancing the OTR to achieve faster growth rates, higher cell densities, and ultimately higher productivity. Implicit from Equation (2) is that several approaches may be taken to do this, including the manipulation of C_L_*, *a*, or *k_L_*. All of the fed-batch studies use very high stirring rates to enhance the OTR by increasing both the tortuosity of the path taken by the bubble and the residence time of the bubble in the liquid phase (gas hold-up, which can be measured as the total liquid volume that is occupied by the gas). While this increases *k_L_*, stirring also helps to increase *a* by breaking up large bubbles. This requires a significant input of mechanical energy to the bioreactor, and is frequently used in conjunction with other techniques. Many studies also increase the driving force by increasing the C_L_*. As previously mentioned, this has included the use of an aeration medium with enriched O_2_ content (as noted in Table 1 and Table 2), as well as pressurization of the bioreactor headspace [79,115,116]. Interestingly, while pressurization increases C_L_*, it has also been shown to have a detrimental impact on *k_L_a* [117]. Other approaches involve increasing *a* using microbubbler devices [118]. Although not used in a PHA production context, Baker et al. (2016) used a microfluidic device to generate bubbles with an average diameter of around 110 μm, and obtained nearly ideal mass transfer (i.e., 90% of the oxygen in the gas phase was delivered to the liquid phase), which is a promising development for industrial biotechnology in general [119].

Other approaches have focused on modifying the medium through the addition of a second phase (liquid or solid) that effectively increases the solubility of O_2_ in the medium. Magnetic functionalized nanoparticles have been used to increase OTR (both *k_L_* and *a*) in bioreactors [120]. However, no reports could be found on the use of functionalized nanoparticles to improve PHA productivity. Two-liquid phase systems, in which small quantities of certain immiscible liquids are dispersed into the aqueous medium, have been reported to enhance OTR [121,122,123,124]. Interestingly, soybean oil (which along with other plant-based oils are high-yielding PHA substrates) has been found to significantly improve *k_L_a* when dispersed in aqueous medium [125,126]. A few studies have investigated two-liquid phase bioreactor systems in PHA production [70,74], but in those cases, n-octane served as the carbon source, and its possible effect on O_2_ solubility was not examined. 

Genetic engineering strategies have also been explored to allow the synthesis of PHA to be more favorable at low DO conditions. Wei et al. [127] cloned several anaerobic promoters upstream of the *phbCAB* operon in *E. coli*. That study found that a strain harboring the promoter for alcohol dehydrogenase (in addition to *phbCAB*) increased the *%_PHB_* of a microaerobic (sealed flasks with no shaking) from 30% to 48% CDM, and this was further improved to 67% CDM when the acetate pathway was deleted. Although not done for the specific purpose of PHA production, Schmitz et al. [128] cloned phenazine redox mediators from *P. aeruginosa* into *P. putida* KT2440. This allowed *P. putida* to maintain oxygen-limited metabolism for two weeks by partial redox balancing in the presence of an anode in low DO environments, and could be a promising development for *P. putida* as a biocatalyst for HCD and large-scale PHA production. 

In summary, maintaining adequate DO at high cell density is a significant challenge for commercial-scale PHA production. As discussed in this section, this is due to a combination of the low solubility of O_2_ in aqueous medium and poor gas-to-liquid mass transfer of O_2_. Although a number of techniques, some of which appear quite promising, may be applied to delay the onset of O_2_-limited conditions and achieve higher cell densities and increased productivities, there are drawbacks to many of these techniques. In particular, the use of purified O_2_ can add significant fixed and operating costs to PHA production [51,116]. Bioreactor pressurization may be a more attractive alternative, but this requires specialized equipment and also makes feeding less straightforward due to the need for bioreactor addition ports that are compatible with pressure. The use of microbubble devices can significantly improve gas-to-liquid mass transfer, but can also require high-energy input [129] and be prone to fouling [130]. Even the high mechanical energy that is required for vigorous agitation and aeration accounts for a large portion of the environmental impact and cost of PHA production [34]. An unfortunate paradox is that many of these developments are not cost-effective to implement in large-scale cultivation systems, which have inherently poor mass transfer characteristics [131,132,133]. Studies that have scaled up fed-batch processes in PHA production are discussed in the following section.

### 2.3. Scale-Up Fed-Batch PHA Production

As discussed above, the PHA productivity of bench-scale fed-batch operations is almost inevitably limited by the bioreactor OTR. Such limitations are exacerbated with increasing scale and represent some of the biggest engineering challenges for bioprocess scale-up. There are many criteria that can be used for the basis of scale-up, including similar geometry, constant impeller tip speed, constant Reynolds number, constant *k_L_a*, circulation time, or constant power input (either gassed or ungassed) per unit reactor volume [134]. Ultimately, all of these criteria are simply different ways of measuring energy requirements, which is the limiting factor in scale-up. For example, according to Lara et al. [133] performing a scale-up from 80 L to 1000 L on the basis of constant circulation time could be done, but would result in a 25-fold increase in power dissipation per unit volume. The lesser power dissipated for mixing in large-scale bioreactors creates non-homogeneous physical and chemical environments for microbes, and generally results in productivity losses of 10–30% at increased scale for many bioprocesses. In a conventional bioreactor design, these gradients usually occur in the vertical direction, since feeding is done from the top and aeration is done from the bottom, and significant hydrostatic pressure near the bottom can also influence gas solubility [135]. This complicates the measurement of important process variables, because the measurement results can be highly dependent on the location of the probes [131]. In order to anticipate some of these challenges, scale is increased by no more than one order of magnitude at a time [134].

Despite these challenges, the ability to demonstrate scalable PHA cultivation techniques is important to reduce costs and improve the economic viability of PHA markets. According to Choi and Lee [51], increasing scale can significantly decrease costs (both operating and capital costs) for production capacities of up to 15,000 tonnes of PHA per annum. In the literature reviewed so far, the majority of the focus in PHA research has been on strategies to improve productivity in lab-scale bioreactors, typically with working volumes of 5 L or less. Studies that have investigated some aspect of scale-up are relatively few, but are shown in Table 4 for a variety of cultivations, including both mcl-PHA and scl-PHA production at scales ranging from at least 100 L to 10,000 L. Relatively few studies have reported this kind of scale-up of at least 100 L. If the bioreactor size is set a lower limit, such as 30–50 L, Table 4 would include quite a few more studies [53,77,84,91,109,136]. 

As indicated in Table 4, scl-PHA productivities of around 1 g L^−1^ h^−1^ have been demonstrated in a few studies at the 300-L (100–150 L working volume) scale [139,140], whereas 0.75 g L^−1^ h^−1^ has been demonstrated for mcl-PHA production at a similar scale [112]. These values are less than half the *Q_v_* values reported in Table 1 and Table 2 for similar processes. This is likely because many of the studies in Table 1 and Table 2 have used purified O_2_ as the aeration medium, which can increase the driving force for oxygen transfer nearly five-fold [146], and is usually impractical in larger scale vessels. This is exemplified by none of the scale-up studies in Table 4, other than one study that used a slightly pressurized headspace, using an O_2_-enriched aeration medium. A recent study performed a *k_L_a*-based scale-up and obtained a *Q_v_* of 1.66 g L^−1^ h^−1^ (P(3-HB-co-4-HB) in a 5000-L bioreactor, which is perhaps the most promising pilot-scale result in PHA production to date [145]. Interestingly, in that same study, the *Q_v_* obtained at 5000 L was slightly higher than that which was achieved at the 1000-L scale, and was significantly better than the result achieved at lab-scale (7.5 L, see Table 4). However, in this study, the estimated *k_L_a* in both the 1000-L and 5000-L bioreactors was similar, and significantly better than the 7.5-L bioreactor. 

Shang et al. [141] evaluated growth and PHB synthesis from *C. necator* using air compared to purified O_2_ as the aeration medium in 5-L and 30-L bioreactors, as well as a 300-L bioreactor where air was the sole aeration medium. Although the focus of that study was the effects of CO_2_ inhibition arising from poor liquid-to-gas mass transfer at lower gas flow rates (used to minimize the use of purified O_2_), the study presents a useful comparison to help deconvolute the effects of increasing the driving force for oxygen transfer by gassing the reactor with pure O_2_. The use of pure O_2_ instead of atmospheric air doubled [*X_t_*] nearly four-fold (from 96 g L^−1^ to 208 g L^−1^) in the 5-L bioreactor and more than tripled [*X_t_*] (from 49 g L^−1^ to 186 g L^−1^) in the 30-L bioreactor. Furthermore, *Q_v_* displayed a two-fold increase from approximately 1 g L^−1^ to 3 g L^−1^ h^−1^ in both the 5-L and 30-L bioreactors. Interestingly, the PHB content was elevated when pure O_2_ was used at both the 5-L and 30-L scale. This is somewhat unexpected, since PHB is known to be synthesized from glucose in low-DO environments [147,148,149]. Oxygen limitation became very prevalent at the 300-L scale, which achieved two-fold less biomass than the 30-L scale, and four-fold less biomass than the 5-L scale when air was used as the sole aeration medium. 

It is worthwhile mentioning that Shang et al. [141] concluded that there was a significant effect of CO_2_ inhibition on both growth and PHA synthesis in *C. necator*. Follonier et al. [115] also evaluated the effect of elevated CO_2_ concentrations in a pressurized bioreactor system, and concluded that dissolved CO_2_ concentrations of up to 540 mg L^−1^ did not negatively affect mcl-PHA synthesis, but did result in decreased μ in cultures of *P. putida* KT2440. Both of these studies suggest that the buildup of CO_2_ from liquid-to-gas mass transfer limitations also merit consideration during process scale-up, since they have been shown to negatively impact both growth and PHA synthesis. 

## 3. Continuous and Semi-Continuous Bioreactor Systems

In continuous culture, fresh medium is constantly supplied to the bioreactor, and a portion of the culture is removed at the same rate: the dilution rate (*D*). The conditions inside the reactor (substrate, cell, and product concentrations) remain at steady state, and because of this, continuous cultures are often referred to as chemostats, which is short for “chemical environment is static” [150]. Steady-state operation has a number of advantages. It is an attractive platform to study bioprocess physiology and implement control strategies due to the constant growth rate, and is less laborious to operate and maintain once steady-state operation is reached [15,150,151]. Steady-state operation also circumvents the major challenge of the optimal control of fed-batch strategies, which involves predicting growth rates over time and under highly dynamic conditions [50].

However, the main reason that continuous bioreactor operations may be of interest is that they can theoretically achieve higher average productivity (over time) in comparison to discontinuous processes [152]. This is done by maintaining the culture at a moderate but constant growth rate for prolonged periods and avoiding the non-productive downtime that is required for harvest, cleaning, preparation of new medium, sterilization, cooling, and culture lag in a discontinuous process [15,150]. Ultimately, higher productivities could result in a smaller reactor volume requirement, less capital investment (for both the production and downstream processing requirements), lower operating costs, and would avoid some of the typical mass transfer issues associated with the scale-up of discontinuous processes, which have already been discussed [150,152,153,154]. Furthermore, the manipulation of chemostat cultures has also been shown to reproducibly tailor monomer composition [153,155,156] and *M_w_* [157], giving the process considerable flexibility.

Despite the significant upsides of continuous cultivation processes, the experimental values for PHA productivities have not reached the levels that have been reported for fed-batch cultivation. [153]. There are a number of other challenges with continuous culturing that have perhaps limited its industrial application. These include: (1) the higher risk of infection due to a large number of fittings and pumping processes; (2) the risk of back-growth into the feed medium reservoir; (3) maintaining genetic stability of the production strain over many generations; and (4) cell washout as the *D* approaches μ_max_ [50,158,159].

### 3.1. Continuous Processes in PHA Production

The *D* is an important parameter in continuous systems, and is defined as the quotient of the bioreactor working volume to the incoming flow rate (h^−1^). This can have a significant impact on μ, *%_PHA_*, and hence *Q_v_* [160,161]. Unless cell retention techniques are employed, the *D* is equal to both the hydraulic retention time and solids retention time, and must be lower than μ_max_ to prevent cell washout or wasting the carbon source (which unless recycled must be subject to post-treatment to remove the biochemical oxygen demand of the waste effluent). On the other hand, μ must be maintained high enough to allow productive growth, keep toxic substrates diluted, and remove any accumulation of toxic end products.

It must be acknowledged that the application of continuous culture for PHA production is a significant challenge. In general, PHA synthesis is not growth-associated, and cannot therefore be simultaneously optimized (along with growth) in a reactor operating at steady state [50,150]. This may be the reason that continuous cultures are often applied as a tool to study physiology rather than an operating strategy to advance PHA productivity. One solution has been to implement a two-stage process in which the two phases (growth and PHA synthesis) are separated spatially by adding a second reactor in-series, where conditions favorable to PHA accumulation (i.e., high C/N or C/P ratios) can be implemented. This has been done in multiple instances with promising results [153,162,163]. One study producing PHB from glucose using *C. necator* has used a cascade of five bioreactors in series (i.e., to simulate plug flow) in order to optimize *%_PHA_* [164]. 

These, and other past studies of PHA production (both mcl-PHA and scl-PHA) in continuous cultures are summarized in Table 5. It is worth noting that Ramsay et al. [159] carried out the original study of mcl-PHA production in continuous culture as a way to circumvent the toxicity effect of related substrates using *P. putida* GPo1 grown on octanoate. While this study showed a relationship of decreased *%_PHA_* with increased *D*, *%_PHA_* was low, and ultimately, it was concluded that a fed-batch strategy should be pursued. This group of researchers subsequently developed high-productivity fed-batch strategies (Table 1).

Reviewing Table 1 and Table 2 reveals that *Q_v_* (for both mcl-PHA and scl-PHA) is significantly lower in continuous processes (to date) than in fed-batch processes. However, it is important to note that fed-batch productivities are always reported on a time-scale from inoculation to harvest. To be able to compare these values with those in Table 6, productivity should be amortized over time from one harvest to the next, factoring in the downtime for cleaning, preparation, sterilization, cooling, and preparation of the inoculum for the subsequent cultivation. Follonier et al. [115] investigated the effects of increased pressure on mcl-PHA production from a combination of octanoic and undecenoic acid in a chemostat culture. Although a reasonable productivity was obtained (Table 5), it was estimated that with some further optimization to increase the OUR (higher aeration), the mcl-PHA productivity in this system could be pushed to as high as 11 g L^−1^ h^−1^, assuming O_2_ to be the only limiting factor. While this number would indeed be a remarkable development, to our knowledge, this has not yet been done.

### 3.2. Cyclic/Repeated (Semi-Continuous) Processes Culture in PHA Production

Repeated or cyclic batch and fed-batch processes could also be adopted as a method to increase *Q_v_* by decreasing the non-productive downtime between batches. Although still a discontinuous process, the growth and PHA production phases can be accomplished in a single bioreactor operated under transient conditions. With this type of operation, a portion of the reactor is decanted and refilled with fresh medium to restart the process immediately after the end of the last cycle [47]. This principle is similar to sequencing batch reactors (SBRs), which have become a popular culture selection tool with subsequent scl-PHA production using mixed microbial cultures (MMCs), allowing for the quick and easy manipulation of process conditions to favor PHA production [40]. Although developed primarily as a selection tool for robust scl-PHA-producing populations from MMCs, they may also be a reliable method for producing mcl-PHA effectively using pure cultures. Although harvest is done at intervals (i.e., discontinuously), there is still a considerable time-saving advantage by using the remaining broth as a seed for the subsequent reactor, eliminating the time required to clean, sterilize, and prepare new inoculum [173]. Several instances of this type of process are shown in Table 6, and some have reported highly competitive productivities. However, in several cases, only a few cycles were done [169,171], and the cultures did not appear to achieve a steady state. Ideally, more prolonged operation would be required to take advantage of the time saved by using a cyclic approach. 

Since waste carbon sources are usually diluted (lactose in whey solution, for example), often these cyclic processes are employed in conjunction with a cell-retention technique (typically an external membrane module) to overcome the volumetric limitation imposed by feeding a diluted carbon source [168,175,176]. This is generally not the case with the studies reported in Table 1 and Table 2. Cell-retention techniques (i.e., a membrane filter) allow the decoupling of the solids’ retention time from the *D*, meaning that cells can be exposed to more carbon and thus accumulate higher *%_PHA_*, while avoiding washout with the effluent. As shown, some of these studies have reported cell densities approaching 150–200 g L^−1^, with PHB content of up to 87% CDM.

### 3.3. Sterility Challenges

One of the challenges of semi-continuous or continuous operation is the increasing risk of contamination [178]. Although pure cultures may be preferred in PHA production because of the reproducibility of the process and products, the cost of sterilization processes is one of the main disadvantages compared with mixed culture PHA production [179]. However, if an environment can be established that precludes or minimizes the growth of competitors, then the lack of a need for sterilization could be a cost-saving measure for pure culture production as well. Such techniques are used in PHA production with MMCs, in which feast-and-famine cycles (aerobic dynamic feeding) are used to select for PHA-producing bacterial populations, because the ability to store carbon gives them a competitive advantage under starvation conditions [180]. Tan et al. [181] isolated a halophilic bacterium (*Halomonas bluephagenesis* TD01) from a salt lake in China. This organism was cultured under non-sterile conditions (37 °C, pH 9.0), and produced 80 g L^−1^ CDM containing 80% PHA in 56 h using a fed-batch method. In a two-stage continuous bioreactor, the steady-state concentration of biomass in the first reactor was 40 g L^−1^ with 60% PHB content, and PHB content rose to 70% CDM after being transferred to the second stage with nitrogen-limiting conditions. In an uninoculated blank study, 3.5 g L^−1^ of cellular contaminants was observed after 48 h, but no contaminants could be detected in the presence of *H. bluephagenesis TD01*. Subsequently, a genetically modified derivative of this strain (TD40, with the succinate semi-aldehyde dehydrogenase deleted) was cultivated in fed-batch mode under non-sterile conditions at the 5000-L scale [145]. In that study, [*X_t_*] of nearly 100 g L^−1^ were achieved along with P(3-HB-co-4-HB) productivities of 1.66 g L^−1^ h^−1^ (see Table 4). 

In a similar study, Yue et al. [172] were able to maintain relatively steady biomass and PHB concentrations, with no evident contamination using wild-type and recombinant *H. campaniensis* LS21 in a 65-day open process that was periodically fed a mixture of fats, proteins, and cellulose-based substrates designed to model kitchen waste. Lillo and Rodriguez [166] made use of the salt-tolerant properties of the haloarcula *Hfx. mediterranei* ATCC 33500 to maintain a continuous culture for three months under non-sterile conditions with stable PHB production. Other studies have employed methanotrophs [182] as well as thermophilic strains [171] for the production of PHA under non-sterile conditions. 

## 4. Discussion and Conclusions

The widespread use of PHAs in biopolymer applications is currently prevented by their high production cost [34], the cost of downstream processing [29], as well as the questionable life cycle-based performance [32]. The upstream production costs can largely be broken down into the cost of the carbon source [39], the cost for mechanical agitation and the aeration of the bioreactor itself [34], as well as the capital and operating costs that are required to maintain a sterile environment [178]. Therefore, the development of efficient cultivation strategies is an important milestone toward producing PHAs in a cost-effective manner. In this review, we have attempted to summarize the bioreactor operational strategies that are used to target high-productivity PHA production using pure cultures. The paragraphs that follow are a summary of the findings. 

Fed-batch strategies appear to be the most popular high-productivity cultivation method, and boast some of the highest reported *Q_v_* values for both mcl-PHA and scl-PHA production. Many earlier studies, particularly from the mid-1990s in scl-PHA production and perhaps 10–15 years later in mcl-PHA production, report some of the highest known *Q_v_* values to this day. However, recently, we have seen a shift away from those studies that simply target reporting the highest known *Q_v_*, perhaps because many of those studies were done under conditions that were only possible at the lab-scale and unrealistic for pilot-scale and industrial-scale bioreactors. These conditions include the use of highly refined carbon substrates and increasing the driving force for oxygen transfer through sparging with O_2_-enriched air.

A look at more recent studies shows a shift toward addressing these issues. Firstly, several recent studies have achieved reasonably good results using more realistic waste carbon substrates, including agricultural residues [98,108], waste glycerol [99,183], VFAs [61,62,96,101], and plant oils [94,102]. Secondly, many of these studies are doing so without use of O_2_-enriched air, which can significantly improve productivity, but also adds significant expense, and is therefore not generally recommended [51,139]. This is exemplified in this review; one study from over 20 years ago used a 50-L bioreactor with pure O_2_ [53], but no such recent studies of scaled-up systems with working volumes of approximately 100 L or greater were found. This suggests that ongoing developments for pilot-scale and industrial-scale PHA production operations should rely on efficient cultivation strategies that are not dependent on pure O_2_ usage. This is currently being addressed at the lab scale, and several promising high-productivity cultivation systems have been developed for both mcl-PHA and scl-PHA production that use only air [61,62,97,103,176] as well as in industrial-scale bioreactors [139,145]. Although other methods for increasing the driving force of oxygen transfer are available, including pressure [115], microbubble devices [118], and microfluidic devices [119], they have not yet been applied on a large scale. 

Time is a significant factor in advancing PHA productivities. While the preferred mode of operation in PHA production certainly appears to be fed batch, continuous processes may have higher average productivity over prolonged operation due to the lack of downtime between batches. Continuous cultures of both scl-PHA and mcl-PHA have resulted in productivities in excess of 1 g L^−1^ h^−1^, but little process development has occurred in this area within the last eight years, which is a problem that was also identified in a 2015 review of strategies for large-scale PHA production, and appears to remain unaddressed at present [45]. 

Cyclic/repeated processes also use time as a factor to advance productivity, although not as effectively as fully continuous bioreactors. There have been a number of promising developments in this area within the last 10 years. Within this context, cell retention techniques can be employed to overcome the volumetric limitation that comes from feeding waste carbon streams in which the substrates are often diluted. When these cell-retention techniques are employed, some studies have achieved productivities in excess of 3 g L^−1^ h^−1^ from waste substrates [168] or diluted carbon streams intended to represent waste streams [176]. While these processes have recently shown great promise, they were generally not operated long enough to take advantage of the time saved from the minimal downtime in a cyclic process. Finally, the capital and operating costs of sterilization are being considered, as is indicated by a recent review of non-sterile biotechnological processes [184]. This is particularly important for continuous and semi-continuous bioreactor systems, which are especially susceptible to contamination [50], but is also being considered in fed-batch cultivations by using halophiles and thermophiles [171,172,181]. 

In summary, with the exception of the lack of process development for full continuous bioreactor systems, the overall trends toward scaling-up, utilizing available inexpensive waste carbon streams, and non-sterile cultivations are promising. The recent work of Ye et al. [145] perhaps best represents the culmination of these technologies, and establishes what is, in our view, a new benchmark to give optimism to the industry: industrial-scale PHA production with high *Q_v_* (1.7 g L^−1^ h^−1^) from an available waste carbon source (corn steep liquor) under non-sterile conditions. These and other similar developments keep PHAs a relevant and worthwhile pursuit for novel biopolymer applications. 

## Figures and Tables

**Table 1:** Chronological summary of process developments for high-productivity fed-batch bioreactor systems in medium-chain length polyhydroxyalkanoate (mcl-PHA) research.

Ref.	Organism	Substrate; Limitation	[*X_t_*] (g L^−1^)	*%_PHA_* (% CDM)	*Y_PHA/S_* (g g^−1^)	*Q_v_* (g L^−1^ h^−1^)	C_L_*	Contribution
[70]	*P. putida* GPo1	Octane; N-limited	37.1	33	-	0.25	Air	First HCD process in two-liquid phase media
[71]	*P. putida* GPo1	Octane; N-limited	40	26	-	0.34	?	Economic evaluation of mcl-PHA production systems
[63]	*P. putida* BM01	OA + glc; N, O_2_-limited	35.9	65.6	0.4	0.92	Air	Enhanced *Y_PHA/OA_* by co-feeding glucose
[72]	*P. putida* GPo1	OA	47	55	0.31	0.54	Air	Studied granule morphology in vivo during fed-batch cultivation
[73]	*P. putida* KT2442	Octanoate; N-limited	51.5	17.4	-	0.41 ^a^	+O_2_	Closed-loop fed-batch control strategies based on online gas chromatography (GC) measurements
[74]	*P. oleovorans*	Octane; O_2_-limited	112	5	-	0.09	+O_2_	Highest [*X_t_*] from octane, but found to be inversely proportional to *%_PHA_*
[75]	*P. putida* KT2442	Oleic acid; P-limited	141	51.4	-	1.91	+O_2_	Highest known [*X_t_*] and highest *Q_v_* from oleic acid
[76]	*P. putida* GPo1	OA; N-limited	63	62	-	1	Air	Highest *%_PHA_* (75% CDM) did not correspond to max. *Q_v_*
[69]	*P. putida* IPT046	Glc + fructose; P-limited	50	63	0.19	0.8	Air	Highest *Q_v_*, *%_PHA_* from glucose in a native strain
[55]	*P. putida* KT2440	NA; C-limited	56	67	0.6	1.44	+O_2_	mcl-PHA accumulation under C-limitation
[48]	*P. putida* KT2440	NA; C-limited	109	63	-	2.13	+O_2_	Highest *Q_v_* yet reported for mcl-PHAs
[77]	*P. putida* KT2440	Corn oil LCFAs, P-limited	103	28.5	-	0.61	+O_2_	High *Q_v_* from mixed LCFA substrate
[78]	*P. putida* KT2440	NA + UDA; C-limited	48.1	55.8	0.5	1.09	+O_2_	Control of PHA monomers through feeding
[56]	*P. putida* KT2440	Glc + NA; C-limited	71	56	0.66	1.44	+O_2_	Improved *Y_PHA/NA_* by co-feeding glucose
[59]	*P. putida* KT2440	Glc + NA; C-limited	71.4	75.5	0.78	1.8	+O_2_	Used acrylic acid as a β-oxidation inhibitor to obtain elevated C9 content
[62]	*P. putida* CA-3	Butyric acid + DA; P-limited	90	65	0.61	1.63	Air	First use of VFA feedstock, highest *Q_v_* without the use of enriched air
[61]	*P. putida* KT2440	Glc + NA; no limitation	102	32	0.56	0.95 ^b^	Air	Highest [*X_t_*] without enriched air
[67]	Recombinant *P. putida Δgcd*	Glc; C-limited	61.8	67	-	0.83	+O_2_	Highest *%_PHA_*, *Q_v_* from glucose
[60]	*P. putida* KT2440	DA, acetic acid, glc (5:1:4); C-limited	75	74	0.86	1.16	+O_2_	Highest *Y_PHA/S_*
[79]	*P. putida* KT2440	Oleic acid (80%); N-limited	125.6	54.4	0.7 ^c^	1.01	Press. (0.3 bar)	Improved *Y_PHA/S_* coupled to anabolism

^a^ Final *Q_v_* reported at 42.75 h, whereas maximum *Q_v_* reported as 1.18 g L^−1^ h^−1^ at 23 h; ^b^ 2.85 g L^−1^ h^−1^ reported only during 11 h of PHA accumulation phase when supplied with nonanoic acid. Factoring in the 21-h growth phase results in a *Q_v_* of 0.95 g L^−1^ h^−1^; ^c^ *Y_PHA/S_* reported yield on a C-mol C-mol^−1^ basis. Abbreviations (not already defined): C_L_*—equilibrium oxygen concentration, which may be enhanced using O_2_-enriched air (+O_2_) or elevated pressure (press.); OA, octanoic acid; glc, glucose; NA, nonanoic acid; DA, decanoic acid; UDA, undecenoic acid; LCFAs, long-chain fatty acids; VFA, volatile fatty acids. The dash line in the Y_PHA/S_ column indicates where values were not reported. The question marks indicate where it was not explicitly stated if aeration was done with atmospheric air, purified O_2_ or some other means of increasing driving fore for oxygen transfer. Note: *P. putida* GPo1 formerly known as *P. oleovorans* (strain ATCC 29347).

**Table 2:** Chronological summary of process developments for high-productivity fed-batch bioreactor systems in short-chain length (scl)-PHA research.

Ref.	Organism; (Product ^a^)	Substrate; Conditions	[*X_t_*] (g L^−1^)	*%_PHA_* (% CDM)	*Y_PHA/S_* (g g^−1^)	*Q_v_* (g L^−1^ h^−1^)	C_L_*	Contribution
[80]	*Methylobacterium extorquens*	MeOH; N-limited	233	64	0.2	0.88	+O_2_	Highest *%_PHB_* obtained using a methylotroph
[81]	*E. coli* XL1-Blue	Glc; O_2_-limited	116.6	76	-	2.11	+O_2_	First HCD fed batch using recombinant *E. coli* for PHB production
[57]	*Cupriavidus necator* NCIMB 11599	Glc; N-limited	164	76	0.3	2.42	Air	Timing of N-limitation and maintaining a residual [glc] of 10–20 g L^−1^ important
[82]	*Methylobacterium organophilum*	MeOH; K-limited	250	52	0.19	1.86	+O_2_	Highest [*X_t_*] obtained using a methylotroph
[83]	*M. extorquens ATCC 55366*	MeOH; N-limited	114	46	0.22	0.56	Air	Highest [*X_t_*], *Q_v_* from a methylotroph without using purified O_2_
[54]	*A. lata* DSM 1123	Sucrose; none	142	50	-	3.97	+O_2_	First HCD process using *A. lata*; Highest *Q_v_* without nutrient limitation
[84]	*C. necator* NCIMB 11599	Glc; P-limited	281	83	0.38	3.14	?	Highest [*X_t_*] obtained in a fed-batch system for PHA production
[49]	*A. lata* DSM 1123	Sucrose; N-limited	111.7	88	0.42	4.94 ^b^	+O_2_	Highest *Q_v_* reported in PHA production
[53]	*E. coli* XL1-Blue	Glc; O_2_-limited	204.3	77	0.28	3.2	+O_2_	Highest [*X_t_*] obtained using *E. coli*
[85]	*E. coli* XL1-Blue	Glc; not stated	194.1	73	-	4.63	?	Highest *Q_v_* reported using *E. coli*
[86]	*E. coli* XL1-Blue	Glc; not stated	149	70	-	2.4	+O_2_	
[87]	*E. coli* CGSC 6576	Whey; not stated	87	80	0.33	1.4	+O_2_	First use of a waste industrial substrate for PHB production using *E. coli*.
[58]	*Aeromonas hydrophila;* PHB-co-PHHx	Oleic acid; P-limited	95.7	45	0.51	1.01	+O_2_	First HCD process for PHB-co-PHHx production
[88]	*C. necator* NCIMB 11599	Glucose; P-limited	208	67	-	3.1	+O_2_	Maintaining residual glc at 9 g L^−1^ gave highest *Q_v, PHB_*
[89]	*Halomonas boliviensis* LC1	MSG; N-limited	23	90	-	1.15	Air	Demonstrated viability of *H. boliviensis* for HCD production of PHB
[90]	*C. necator* DSM 529	Crude glycerol, N-limited	76	50	0.34	1.1	+O_2_	Demonstrated high *Q_v, PHB_* obtained using crude glycerol
[91]	*Zorbellella denitrificans* MW1	Glycerol; 20 g L^−1^ NaCl	81.2	66.9	0.25	1.09	Air	Highest known *Y_PHB/S_* using crude glycerol as the sole carbon source
[92]	*C. necator* A-04; P(3-HB-co-4-HB)	Fructose + BDO; C/N ratio of 4 then 200	112	64	-	0.76	Air	Highest obtained [*X_t_*] in P(3-HB-co-4-HB) production
[93]	*Burkholderia sacchari* IPT 189	Sucrose; N, O_2_ limited	150	42	0.22	1.7	?	Utilized an airlift bioreactor (instead of STR) for HCD production of PHB
[94]	*C. necator* DSM 545	Soybean oil; N, P, metals limiting	83	80	0.85	2.5	?	Highest *Q_v_* obtained from edible oils as the sole carbon source.
[95]	Recombinant *C. necator*; PHB-co-PHHx	Palm oil; N-limited	139	74	0.78 ^c^	1.07	+O_2_	Amongst the highest [*X_t_*] and *Q_v_* for production of PHB-co-PHHx using recombinant *C. necator*
[96]	*C. necator* DSM 545	Butyric acid; P-limited	46.7	82	0.62 ^d^	0.57	Press (75 mbar)	Demonstrated highest [*X_t_*], *%_PHA_*, and *Qv* for *C. necator* grown on butyric acid and favorable impact of coupling residual growth and PHB synthesis on *Q_v_*
[97]	*Bacillus megaterium* BA-019	Sugarcane molasses; C/N = 10	73	43	-	1.73	Air	Highest obtained *Q_v_* for PHB production using *B. megaterium*
[98]	*B. sacchari* DSM 17165	WSH; P-limited	146	72	0.22	1.6	Air	Highest *Q_v_* from a waste agricultural residue
[99]	*C. necator* DSM 7237; PHBV	Crude glycerol and levulinic acid with SFM; C/N = 17.05	27.9	74.5	0.34	0.27	Air	Among the highest *%_PHBV_* and [*X_PHBV_*] produced using levulinic acid as the precursor
[100]	*C. necator* DSM 545	Glc; N-limited	128	76	0.24	2.03	+O_2_	Developed fed-batch control strategy independent of the carbon source
[101]	*C. necator H16*; PHBV	Mixed VFAs; N-limited	112.4	83	-	2.13	+O_2_	Highest [*X_t_*], *Q_v_* in production of PHB with poly(3-hydroxyvalerate) (PHBV)
[102]	*C. necator* Re2058/pCB113; PHB-co-PHHx	SPO; N-limited	88.3	57	0.5	1.1	Air	Demonstrated efficacy of SPO as a substrate for high-Qv production of PHB-co-PHHx
[103]	*B. sacchari* DSM 17165; P(3-HB-co-4-HB)	Saccharose + GBL; N-limited	74.6	72	0.08	1.87	Air	Highest known *Q_v_* for the product P(3-HB-co-4HB)

^a^ Product is poly(3-hydroxybutryate) (PHB) unless otherwise specified; ^b^ *Q_v_* values reported at time of harvest (20 h), highest *Q_v_* of 5.13 g L^−1^ h^−1^ observed at 16 h; ^c^ Values reported during polymer synthesis phase; ^d^ *Y_PHA/S_* reported as C-mol C-mol^−1^. Abbreviations: GBL, γ-butyrolactone, BDO, 1,4 butanediol; MeOH–methanol; WSH, wheat straw hydrolysate; SFM, sunflower meal hydrolysate; SPO, sludge palm oil; STR, stirred-tank reactor.

**Table 3:** Various feeding strategies used to obtain HCD cultures in mcl-PHA production.

Ref.	Feeding Strategy	Results
[48]	Exponential feeding of NA: (1) From 0 to 9 h starting at μ = 0.25 h−1 followed by a linearly decaying feeding rate over the next 21 h (2) Quadratic decay in feeding rate (5 to 15 h) with initial μ = 0.45 h−1 followed by a constant feed rate (8.75 g NA L−1 h−1)	(1) [Xt] = 90 g L−1 CDM with 65% PHA 30 h, Qv = 1.9 g L−1 h−1 in 30 h. (2) [Xt] = 109 g L−1 CDM with 63% PHA in 30 h, Qv = 2.13 g L−1 h−1.
[60]	Exponential feeding of a mixture of DA, acetic acid, and glc (5:1:4) at μ = 0.15 h−1 for 23 h followed by constant feeding rate of 5 g of substrate L−1 h−1 until 40 h.	[Xt] = 75 g L−1 CDM containing 74% PHA in 40 h, Qv = 1.16 g L−1 h−1.
[61]	Several exponential glc feeding strategies evaluated: (1) At a constant specific growth rate of 0.25 h−1 until dissolved oxygen (DO) limitation (2) At μmax (0.67 h−1) for 0 to 9 h followed by constant feeding when DO became limiting (3) At μmax (0.67 h−1) for 0 to 9 h followed by a linearly increasing feed rate when DO became limiting (4) Same as in (3) but with a constant feed of NA (98 g h−1) imposed at 21 h (65 g L−1 CDM)	(1) [Xt] = 53 g L−1 CDM in 22 h. Ended by DO limitation causing glc accumulation. (2) [Xt] = 43 g L−1 CDM in 18 h. No DO limitation but C limitation slowed growth. (3) 102 g L−1 CDM in 33 h, limited by DO. (4) [Xt] = 98 g L−1 containing 32% PHA obtained in 32 h.
[112]	(1) Cells allowed to grow for the first 4 to 5 h, pH adjusted with NH4OH (2) At 6 to 7 h, 0.5 mM of ammonium octanoate and 0.05 mM of MgSO4 were fed per hour for 5 h, then feeding exponentially increased to 5 mM of ammonium octanoate and 0.5 mM of MgSO4 per hour for 5 h (3) 1.8 mM of ammonium octanoate and 0.27 mM of MgSO4 were added per hour	[Xt] = 53 g L−1 CDM containing 50% PHA obtained in 48 h, Qv = 0.76 g L−1 h−1.
[67]	Phase 1: batch operation until 12 h when 20 g L−1 glc initially consumed Phase 2: exponential feeding with μ = 0.2 h−1 until 21 h (50 g L−1 CDM), DO limitation causes glucose accumulation Phase 3: glc fed in response to DO rise above set point to maintain 30 to 35 g L−1	[Xt] = 61.8 g L−1 CDM containing 67% PHA in 50 h for a Qv of 0.83 g L−1 h−1.

**Table 4:** Chronological summary of studies that have examined scale-up of various processes for scl-PHA or mcl-PHA production.

Ref.	Organism, Product	Conditions	WV (TV)	[*X_t_*]	*%_PHA_*	*Y_PHA/S_*	*Q_v_*	C_L_*
			(L)	(g L^−1^)	(% CDM)	(g g^−1^)	(g L^−1^ h^−1^)	
[137]	*Aeromonas hydrophila* 4AK4, P(HB-co-HHx)	Glc during growth and lauric acid during P-limited accumulation phase	10,000 (20,000)	50	50	-	0.54	Air
[138]	*P. putida* GPp104, P(3-HB-co-3-HV-co-4-HV)	DO-stat feeding of gluconic acid during growth, with levulinic acid during the N-limited accumulation phase	500 (650)	19.7	50	-	-	Air
			25 ^a^	25	50	-	-	Air
[139]	Recombinant *E. coli*, PHBV	pH-stat feeding of glc + oleic acid + propionic acid. *k_L_a* = 108 h^−1^ in 300 L bioreactor and *k_L_a* = 396 h^−1^ in 30 L bioreactor	100 (300)	29.6	69	-	1.06	Air
			10 (30)	42.2	70.1	-	1.37	Air
[140]	Recombinant *E. coli* CGSC 4401, PHB	Whey solution containing 200 g L^−1^ lactose fed via pH-stat method	150 (300)	30	67	-	1.01	Air
			10 (30)	51	70	-	1.35	Air
[141]	*C. necator* NCIMB 11599, PHB	Glc controlled at 9 g L^−1^ with online glc analyzer	300 ^a^	23.4	36	-	0.18	Air
			30 ^a^	49.2	45	-	1.09	Air
			5 ^a^	96.4	58	-	1.03	Air
[112]	*P. putida* GPo1 mcl-PHA	Exponential followed by constant feeding of OA with N-limited conditions	350–400 (650)	53	50	0.41	0.76	Air
[142]	*Burkholderia cepacia* ATCC 17759, PHB	Biodiesel waste glycerol concentration maintained at 10–40 g L^−1^ with N-limitation	200 (400)	23.6	31	-	0.06	Air
[143]	*C. necator* H16, PHB	Gluconate with N limitation	400 (650)	24.2	65.2	-	0.23	Air
[144]	*Halomonas campisalis* MCMB-1027, PHB	Batch cultivations using maltose with O_2_ limitation after 12 h (1–5%). Scale-up based on constant *k_L_a* of 14.2–18.4 h^−1^	85 (120)	1.3	49.2	0.06	0.03	Press. (0.5 bar)
			8 (14)	1.7	56.2	0.09	0.04	Press. (0.5 bar)
[108]	*P. putida* KT2440, mcl-PHA	Batch growth on grape pomace with subsequent N-limited polymer accumulation on OA and UDA	100 (300)	14.2	41.1	0.79	0.1	Air
[145]	*Halomonas bluephagenesis* TD40, P(3-HB-co-4-HB)	Glucose, GBL, waste corn steep liquor fed with PHA synthesis triggered by N-limitation. Scale-up based on similar reactor geometry and *k_L_a*	3500 (5000)	99.6	60	-	1.66	Air
			700 (1000)	89.5	64	-	1.58	Air
			4 (7.5)	81.4	74	-	1.25	Air

^a^ Cultivations at 25-L scale not stated whether working volume or total volume. Abbreviations: WV, initial working volume; TV, total volume.

**Table 5:** Chronological summary of continuous culture studies in PHA production.

Ref.	Organism, Substrate (Polymer)	Conditions	[*X_t_*] (g L^−1^)	*%_PHA_* (% CDM)	*Y_PHA/S_* (g g^−1^)	*Q_v_* (g L^−1^ h^−1^)	Contribution
[149]	*A. beijerinckii,* glc (PHB)	Single-stage, O_2_-limited with *D* = 0.05 h^−1^	-	45	0.13	-	First application of PHA production in a chemostat.
[165]	*C. necator* DSM 545, glc + propionate, (PHBV)	Single-stage with N-limited conditions *D* = 0.15 h^−1^	-	33	-	-	Demonstrated copolymer production dependent on the ratio of feed components, also first implementation of a two-stage system for improved *%_PHA_*.
[165]	*A. lata* ATCC 29714, sucrose, propionate (PHBV)	Two-stage with N-limitation, *D* = 0.15 h^−1^	-	58	-	-	
[166]	*Hfx. mediterranei DSM 1411*, glc (PHB)	Single-stage with *D* = 0.02 h^−1^, OTR = 0.4 mmol O_2_ L^−1^ h^−1^	3.1	48		0.03 ^a^	First continuous PHA production using archaea, and first under non-sterile conditions.
[159]	*P. oleovorans* ATCC 29347, OA (mcl-PHA)	Single-stage, various *D* and C/N ratios tested (*D* = 0.24 h^−1^ shown)	4.2	13	0.09	0.14 ^a^	First mcl-PHA production in continuous culture. Demonstrated little effect of feed C/N ratio on *%_PHA_* at *D* = 0.24 h^−1^, and showed some dependence of monomer composition on *D*.
[158]	*P. putida* GPo1, n-octane (mcl-PHA)	Single-stage, *D* = 0.2 h^−1^ NH_4_ limiting	11.6	23	-	0.58	First continuous study with two-liquid phase medium and the first to use a continuous platform for enhanced *Q_v_*. Demonstrated effect of *D* on *%_PHA_*.
[157]	*C. necator*, fructose + pentanoic acid (PHBV)	Single-stage, *D* = 0.17 h^−1^, NH_4_-limiting	-	-	-	0.31	First study to use continuous scl-PHA production as a platform for improved *Q_v_*. Showed effect of the dilution rate on HV content.
[154]	*P. putida* KT2440, oleic acid (mcl-PHA)	Single-stage, O_2_ limited (DO < 15% AS) with *D* = 0.1 h^−1^	30	23	-	0.69	Highest steady-state [*X_t_*] reported for a continuous system in mcl-PHA production.
[71]	*P. putida* GPo1, octane (mcl-PHA)	Single-stage, N limited with *D* = 0.2 h^−1^	12.4	30	0.63	0.74	Highest *Q_v_* reported for a single-stage continuous PHA production system.
[167]	*P. putida* GPo1, OA (mcl-PHA)	Single-stage, *D* = 0.05–0.4 h^−1^, C/N ratio = 5–25 (mol mol^−1^)	-	56.1	0.25	-	First study exploring dual nutrient limited growth. Results reported at *D* = 0.1 h^−1^.
[163]	*C. necator*, glc (PHB)	Two-stage, *D* = 0.075 h^−1^ and with N-limiting conditions in second stage	42	72.1	0.36	1.23	Highest [*X_t_*] and *Q_v_* in continuous culture for PHA production. Maximum *%_PHA_* and *Y_PHA/S_* with *D* = 0.075 h^−1^ in S2.
[162]	*P. oleovorans ATCC 29347*, octane (mcl-PHA)	Two-stage (S1: *D* = 0.21 h^−1^ and S2: *D* = 0.16 h^−1^)	71.4	63	-	1.06	Highest mcl-PHA productivity to date in a continuous bioreactor system.
[155]	*P. putida* GPo1, OA, UDA (mcl-PHA)	Single-stage, *D* = 0.1–0.4 h^−1^, dual C- and N-limitation	1.75	23	-	0.08	Monomer composition shown to be a function of *D* and not the C/N ratio. Preference for the incorporation of aliphatic monomers at lower *D*. Results reported for C/N = 23 and *D* = 0.1 h^−1^.
[153]	*P. putida* GPo1, fatty acids, (mcl-PHA)	Two-stage (S1–octanoic acid, S2–undecenoic acid), *D* = 0.1 h^−1^ for both S1 and S2, dual C and N limitation	1.53(S2)	52.4 (S2)	-	0.08 ^a^	Synthesis of two mcl-PHA copolymers when fed different fatty acids in S1 (OA) and S2 (UDA). Results shown for *D* = 0.1 h^−1^ and C/N = 19.1.
[164]	*C. necator* DSM 545, glc (PHB)	Five CSTRs with *D* = 0.13–0.17 h^−1^; S1 7.5 L growth reactor (DO = 40%) and S2–S5-3.6 L accumulation reactors (DO = 20%)	81 (S5)	77 (S5)	0.29	1.85	First time more than two in-series CSTRs used for PHA production. Highest reported *Q_v_* for any continuous PHA production system.
[115]	*P. putida* KT2440, OA + UDA	High-pressure (7 bar) chemostat with DO = 235%, *D* = 0.15 h^−1^ and C/N = 12.5 g g^−1^	13.8	45.1		0.9	First application of a pressurized chemostat for improved OTR and overall *Q_v_*.

^a^*Qv* calculated on the basis of [*X_t_*] and *%_PHA_*, or [*X_PHA_*], and *D*. Abbreviations: CSTR, continuous stirred tank reactor. Note: *P. putida* GPo1 formerly known as *P. oleovorans* strain ATCC 29347.

**Table 6:** Semi-continuous (cyclic) batch, fed-batch, and cell recycle processes applied in PHA production.

Ref.	Organism	Substrate (Polymer)	Conditions	[*X*_t_] (g L^−1^)	*%_PHA_* *(% CDM)*	*Y_PHA/S_* (g g^−1^)	*Q_v_* (g L^−1^ h^−1^)	C_L_*
[168]	Recombinant *E. coli* CGCS 4401	Whey (PHB)	2.3-L pH-stat fed batch with constant volume and cell recycle via external membrane module (36.5 h total cultivation time)	194	87	-	4.6	O_2_
[169]	*C. necator* NRRL B14690	Fructose (PHB)	Fed batch with 20% volume removal at 27 h and 41 h (two cycles, 68 h total cultivation time)	49	51	0.31	0.42	?
[170]	*Hfx. mediterranei* ATCC 33500	ERB and ECS (1:8) + YE (PHB)	5-L pH-stat repeated fed batch with 90% withdrawal at end of cycle (three cycles, 118 h total cultivation time)	140	55.6	-	3.2	?
[171]	*Chelatococcus* sp. *MW10*	Glucose (PHB)	Fed batch with 20–40% volume removal (two cycles, 265 h total cultivation time)	115	11.8	0.11 ^a^	-	Air
[172]	*Recombinant Halomonas campaniensis* LS21	Mixed (proteins, fats, cellulose) (PHB)	Fed batch with 40 mL of culture removed every 12 h and addition of 2 L of substrate (day 25 and 49) and 0.5 L of seawater on day 7 and 34 (65 d total cultivation time)	69 ^b^	70	-	-	?
[173]	*A. lata* TISTR 1403	Sugar cane juice (PHB)	2-L repeated batch with 90% withdrawal at the end of each cycle (four cycles, 84 h total cultivation time)	4.5	69	0.38 ^c^	0.21	?
[174]	*C. necator* DSM 545	Glucose, fructose (PHB)	5-L repeated fed batch with periodic 25% withdrawal and complete cell recycle via external membrane (eight cycles, 42 h total cultivation time)	61.6	69	-	1.0	Air
[175]	*C. necator* DSM 545	Glucose + propionic acid (PHBV)	Fed batch with cell recycle via an external membrane, with 1 L periodically withdrawn and replaced with fresh medium (52 h total cultivation time)	80	73	-	1.24	Air
[176]	*C. necator* DSM 545	Dilute glucose (PHB)	3-L fed batch, continuously added and removed through external membrane after 18 h of batch operation (36 h total cultivation time)	148	76	0.33	3.1	Air
[177]	*Azohydromonas australica* DSM 1124	Sucrose (PHB)	20% volume removed when sucrose concentration reached 8 g L^−1^, and replenished with fresh medium. Three cycles completed (69 h total cultivation time)	27.9	74	0.59 ^d^	0.29	Air

^a^ Maximum *Y_PHA/S_* reported at end of second cyclic fed batch cycle; ^b^ Maximum [*X_t_*] reported at day 12; ^c^ Maximum *Y_PHA/S_* reported at 18 h; ^d^ Maximum *Y_PHA/S_* reported at 48 h; the dashed line indicates that *Y_PHA/S_* values were not reported. The question marks indicate where the use of air or O_2_-enriched air was not explicitly stated. Abbreviations: ECS, Extruded cornstarch; ERB, Extruded rice bran; YE, Yeast extract.
